# Bacterial Antagonists of Fungal Pathogens Also Control Root-Knot Nematodes by Induced Systemic Resistance of Tomato Plants

**DOI:** 10.1371/journal.pone.0090402

**Published:** 2014-02-28

**Authors:** Mohamed Adam, Holger Heuer, Johannes Hallmann

**Affiliations:** 1 Institute for Epidemiology and Pathogen Diagnostics, Julius Kühn-Institut, Federal Research Centre for Cultivated Plants (JKI), Braunschweig, Germany; 2 Department of Zoology and Nematology, Cairo University, Giza, Egypt; Graz University of Technology (TU Graz), Austria

## Abstract

The potential of bacterial antagonists of fungal pathogens to control the root-knot nematode *Meloidogyne incognita* was investigated under greenhouse conditions. Treatment of tomato seeds with several strains significantly reduced the numbers of galls and egg masses compared with the untreated control. Best performed *Bacillus subtilis* isolates Sb4-23, Mc5-Re2, and Mc2-Re2, which were further studied for their mode of action with regard to direct effects by bacterial metabolites or repellents, and plant mediated effects. Drenching of soil with culture supernatants significantly reduced the number of egg masses produced by *M. incognita* on tomato by up to 62% compared to the control without culture supernatant. Repellence of juveniles by the antagonists was shown in a linked twin-pot set-up, where a majority of juveniles penetrated roots on the side without inoculated antagonists. All tested biocontrol strains induced systemic resistance against *M. incognita* in tomato, as revealed in a split-root system where the bacteria and the nematodes were inoculated at spatially separated roots of the same plant. This reduced the production of egg masses by up to 51%, while inoculation of bacteria and nematodes in the same pot had only a minor additive effect on suppression of *M. incognita* compared to induced systemic resistance alone. Therefore, the plant mediated effect was the major reason for antagonism rather than direct mechanisms. In conclusion, the bacteria known for their antagonistic potential against fungal pathogens also suppressed *M. incognita*. Such “multi-purpose” bacteria might provide new options for control strategies, especially with respect to nematode-fungus disease complexes that cause synergistic yield losses.

## Introduction

Root-knot nematodes (*Meloidogyne* spp.) are among the most damaging sedentary endoparasitic nematodes worldwide. The various species within this genus have an overall host range covering approximately 5500 plant species [1]. The species *Meloidogyne* ***incognita*** which is the most important under economic aspects can infect 1,700 plant species [2]. Root-knot nematodes also interact with fungal pathogens. A nematode-fungus in­teraction was first recorded by Atkinson in 1892, who observed that infection by root-knot nematodes always increased the severity of *Fusarium* wilt [3]. Such interactions often result in a disease complex causing synergistic yield losses [4] as described for root-knot nematodes and soil-borne fungal pathogens like *Thielaviopsis basicola*, *Rhizoctonia solani, Verticillium dahliae* and *Fusarium oxysporum* [5]. Controlling just one of the pathogens might not fully solve the problem. Combinations of nematicidal and fungicidal treatments are possible but not always desired due to their negative impact on the environment and human health. An alternative could be the use of microorganisms with dual antagonism against both the nematode and the fungal pathogen.

Bacteria represent an important group of biocontrol agents and several commercial products are nowadays available to control plant-parasitic nematodes [6] or fungal pathogens [7]. Only few studies previously investigated concomitant effects of bacterial antagonists against fungal and nematode pathogens. Bacterial isolates of the genera *Pseudomonas* and *Streptomyces* were described to control both *V. dahliae* and *M. incognita* [8]. A strain of *Pseudomonas aeruginosa* was found to be antagonistic towards *Meloidogyne javanica* and the fungal pathogens *Macrophomina phaseolina*, *R. solani*, *Fusarium solani*, and *F. oxysporum* [9]. Considering the broad spectrum of microbial antagonists reported over the past decades, different and more efficient microbial antagonists might be around waiting for discovery. The present work focused on bacterial strains The mechanisms of bacteria to antagonize plant-parasitic nematodes include parasitism, pathogenesis, competition, repellence and induced systemic resistance [10]–[13]. Understanding their mode of action will help improving their effectiveness [10].

In the present work, bacterial isolates of the species *Bacillus subtilis, Pseudomonas trivialis, Pseudomonas jessenii*, and *Serratia plymuthica* were selected to study their antagonistic potential against the root-knot nematode *M. incognita* on tomato under greenhouse conditions. All strains have previously shown antagonistic potential towards soil-borne fungal pathogens [14]–[17]. From the first experiment, the top three bacterial strains plus *Rhizobium etli* G12 as positive control were selected for further studies on their mode of action. The objectives of this study were i) to evaluate the biocontrol potential of fungal antagonists towards *M. incognita*, and ii) to investigate their mode of action.

## Materials and Methods

### Bacterial isolates

In total, nine bacterial isolates were tested in various experiments (Table 1). Four bacterial isolates (Sb3-24, Sb4-23, Mc5-Re2, Mc2-Re2) have previously shown *in-vitro* activity against fungal pathogens and *M. incognita* juveniles [17]. Three bacterial isolates (3Re2-7, C48, Ru47) are known antagonists of fungal pathogens [14]–[16]. Finally, the nematode antagonistic bacterium *R. etli* G12 served as positive control and *Escherichia coli* JM109 as negative control, respectively.

**Table 1:** Bacterial isolates used in this study.

Strain	Bacterial species	Isolation source	Pathogen suppressed	Reference	Source a
Sb3-24	*Bacillus subtilis*	Soil	*Verticillium dahliae, Rhizoctonia solani, Fusarium culmorum, Meloidogyne incognita*	[17]	GB
Sb4-23	*Bacillus subtilis*				GB
Mc5-Re2	*Bacillus subtilis*	Endorhiza of chamomile			GB
Mc2-Re2	*Bacillus subtilis*				GB
3Re2-7	*Pseudomonas trivialis*	Endorhiza of potato plants	*Rhizoctonia solani*	[14]	GB
C48	*Serratia plymuthica*	Rhizosphere of oilseed rape	*Verticillium dahliae*	[16]	GB
Ru47	*Pseudomonas jessenii*	Suppressive soil	*Rhizoctonia solani*	[15]	KS
G12	*Rhizobium etli*	Rhizosphere of potato plants	*Meloidogyne incognita*	[37]	RS
JM109	*Escherichia coli*		Non-antagonistic		P

a GB: G. Berg, University of Technology, Graz, Austria; KS: K. Smalla, Julius Kühn-Institut, Braun­schweig, Germany: RS: R. Sikora, Bonn University, Germany; P: Promega, Mannheim, Germany.

### Nematodes

The root-knot nematode *M. incognita* used in all experiments was propagated on tomato (*Solanum lycopersicum*) cv. Moneymaker under greenhouse conditions. For gaining nematode inoculum, eggs were extracted from heavily galled tomato roots. Roots were cut into 1–2 cm pieces, transferred to a 500 ml plastic bottle half filled with a 1.5% chlorine solution and vigorously shaken for 3 min to free the eggs from the gelatinous matrix [18]. The suspension was then thoroughly washed with tap water through a 250 µm aperture sieve, and eggs retained on the 20 µm sieve. To separate hatched second-stage juveniles (J2) from eggs the egg suspension was placed on a modified Baermann dish and incubated at 25±2°C for 7–10 days [19]. Hatched J2 were collected daily and stored at 6°C until further use in the experiments.

### Plants and growing conditions

Tomato cv. Moneymaker was used in all experiments. Tomato seeds were grown in plastic pots containing a mixture of field-soil and sand (1∶1, v:v(. The plants were watered as needed and fertilized weekly with 10 ml of commercial fertilizer (WUXAL® Super NPK fertilizer, 8-8-6 with micronutrients, 2.5 g liter^−1^). Pots were kept in the greenhouse at 25±2°C and 16-h photoperiod.

### Experimental evaluation

Nematode penetration was determined seven days after inoculation by staining the roots with a 1% acid fuchsine solution. Stained roots were kept in the refrigerator overnight to intensify the staining process. Excess acid fuchsine was removed by washing the roots in tap water. Roots were cut into 1 cm pieces and macerated twice for 15 s with a commercial blender (Waring, Torrington, CT, USA) and the number of juveniles in the root suspension was counted at 20 x magnification under a stereomicroscope.

Nematode reproduction was determined 50 days after nematode inoculation by counting the number of galls, egg masses and eggs produced by *M. incognita* on the tomato roots. Roots were gently washed to remove adhering soil. Fresh weights of shoots and roots were taken. Egg masses attached to the roots were stained with a 0.4% cochenille red (Brauns-Heitmann, Warburg, Germany) solution for 15 min. After excessive stain was removed by washing the roots in tap water the number of galls and egg masses was counted. Thereafter, roots were cut in 1–2 cm pieces and transferred into a glass bottle half filled with a 2% chlorine solution. Roots were heavily shaken for 3 minutes and the suspension was then thoroughly washed with tap water through a 250 µm sieve to remove root debris. Eggs collected on a 20 µm sieve were transferred into a glass beaker and counted.

### Experiment 1: Potential of seed-inoculated strains to control *M. incognita*

Seven bacterial strains were investigated for their antagonistic activity against *M. incognita* in pot experiments. Tomato seeds were mixed in a bacterial lawn grown overnight on tryptic soy agar (Merck, Darmstadt, Germany) at 28°C for 24 h until the seed surface was completely covered by bacteria. The treated seeds were left a few minutes under a laminar flow hood for drying, and then each seed was transferred in 11-cm diameter plastic pots containing 400 g of soil watered to field capacity. Pots containing seeds that were treated with cells of strain G12 served as positive control, and pots with *E. coli* treated or untreated seeds served as negative controls. Each treatment was replicated 12 times. Pots were arranged in randomized block design in the greenhouse and kept under the experimental conditions described above. Three weeks later, each pot was inoculated with 1,000 freshly hatched J2 in four holes of 2 cm depth at 3 cm distance from the stem base. The numbers of generated galls and egg masses per plant were counted 50 days after J2 inoculation.

### Experiment 2: Effect of bacterial culture supernatants towards *M. incognita*

As an outcome of experiment 1 the top three bacterial isolates were selected for studying their mode of action: Sb4-23, Mc2-Re2, and Mc5-Re2. Bacterial isolates G12 and *E. coli* served as positive and negative control, respectively. Bacterial cultures were grown from 200 µl pre-culture in 100 ml tryptic soy broth (TSB, Merck, Darmstadt, Germany) for 24 h at 28°C with shaking, and centrifuged at 7500 g for 20 min. Three-week-old tomato seedlings were grown in 7×7×8 cm pots, each containing 300 g soil. The top soil layer (2 cm) was removed. The soil surface was drenched with 20 ml of the respective bacterial culture supernatant or sterile TSB and covered with the previously removed soil. Three days later, a suspension with 1,000 J2 was inoculated into four holes at 2 cm distance from the stem of each plant. Each treatment was replicated ten times and arranged in a randomized block design in the greenhouse. All plants were kept under the experimental conditions described. Fifty days after nematode inoculation the fresh weight and length of shoot and root, and the numbers of leaves, galls, egg masses, and eggs were determined for each pot.

### Experiment 3: Effect of antagonistic strains on repellence of J2

**Figure 1:**
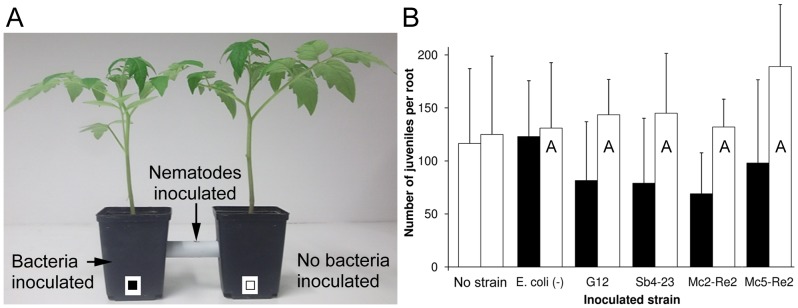
Effect of bacterial antagonists on repellence of *M. incognita* juveniles. Juveniles were attracted by tomato roots and moved from a tube connecting two pots either to the side inoculated with an antagonistic strain or to the opposite side. Controls were inoculated on one side with the not antagonistic strain *E. coli* JM109, or left uninoculated. Juveniles penetrated into the roots were counted on both sides. Error bars represent standard deviations. Different letters indicate significant differences at *P≤0.05* according to Tukey's test (n = 10).

This experiment was conducted using the linked twin-pot chamber as described in a previous study [20]. The two plastic pots of 7×7×8 cm were filled with 300 g soil and connected by a plastic tube of 1 cm diameter and 4 cm length filled with soil (Fig. 1A). Tomato seeds were coated with bacterial cells as described. The treated seeds were grown in the right pot while untreated seeds were grown in the left pot. In the control both pots received untreated seeds. The bacterial culture of these bacterial isolates was prepared following the procedure described above, and then centrifuged at 7,500 g for 20 min. The supernatant was discarded and the resulting pellet was washed then resuspended in sterile tap water. The bacterial density was adjusted to 0.8 at 560 nm, corresponding to 3.2×10^7^ cfu ml^−1^ (Sb4-23), 2.4×10^7^cfu ml^−1^ (Mc2-Re2), 1.8×10^7^cfu ml^−1^ (Mc5-Re2), 1.2×10^7^cfu ml^−1^ (*E. coli*) and 4×10^7^cfu ml^−1^ (G12). Three weeks later, the right pots were inoculated with 10 ml of a bacterial suspension (OD_560_560 = 0.8). The bacterial suspension was added into four holes of a depth of 2 cm around the stem base. After three days, 2,000 J2 in 1 ml water were inoculated through a small hole in the centre of the tube. The hole was sealed with plastic to maintain moisture. Each treatment was replicated ten times. The linked twin-pot chambers were arranged in a randomized block design in the greenhouse and kept under the experimental conditions described. Seven days after nematode inoculation the numbers of J2 penetrated into the roots on both sides of the linked twin-pot chambers were determined.

### Experiment 4: Induced systemic resistance towards *M. incognita*

**Figure 2:**
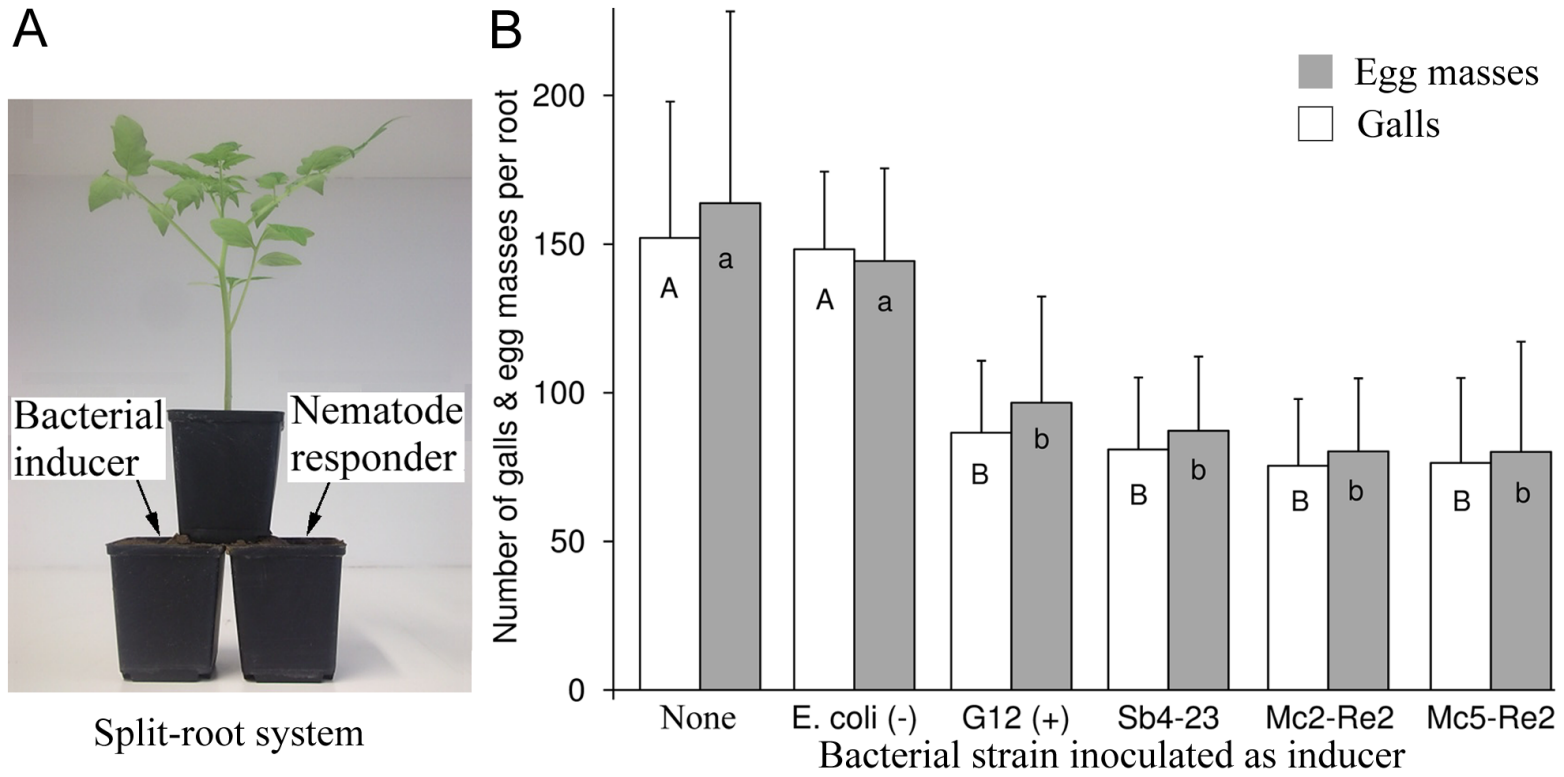
*M. incognita* reproduction affected by bacterial antagonists through induced systemic resistance of tomato. Juveniles and bacteria were inoculated in opposite pots of split root systems. Controls were inoculated with the not antagonistic strain *E. coli* JM109, or left uninoculated. A: Experimental setup of the split root system. B: Mean numbers of galls (white bars) and egg masses (gray bars) counted 50 days after nematode inoculation; error bars represent standard deviations, different letters indicate significant differences at *P≤0.05* according to Tukey's test (n = 10).

Tomato plants were grown in a split-root system as described in a previous study [21]. Three 7×7×8 cm plastic pots were used with one pot placed on top of two pots (Fig. 2A). One tomato seed was placed in the centre of the upper pot half filled with soil. Roots grew through holes in the bottom equally into the two lower pots which were completely filled with soil. After three weeks, one of the two bottom pots termed inducer side was inoculated with 20 ml of a bacterial suspension in tap water (OD_560_560 = 2, corresponding to 8×10^9^ cfu ml^−1^ for strain Sb4-23, 5×10^9^ cfu ml^−1^ for Mc2-Re2, 4×10^9^ cfu ml^−1^ for Mc5-Re2, 1×10^9^ cfu ml^−1^ for *E. coli*, or 1.2×10^10^ cfu ml^−1^ for G12). Plants treated at the inducer side with an equivalent amount of tap water served as control. Three days later, each bottom pot opposite to the inducer side, termed responder side, was inoculated with 1,000 J2. Each treatment was replicated ten times, and arranged in a randomized block design. Fifty days after nematode inoculation galls and egg masses were counted on the roots of the inducer and the responder side.

### Experiment 5: Comparison of the effects by direct and plant-mediated antagonism

In this experiment it was evaluated whether the indirect effect of the bacteria via the plant could fully explain the inhibition of *M. incognita*, or whether co-inoculation in the same pot could enhance the effect through direct antagonism. Three-week-old tomato seedlings grown in the spilt-root systems as described above were divided into three groups: i) plants treated with bacteria on the inducer side and J2 on the responder side, ii) plants kept untreated on the inducer side and treated with bacteria and J2 on the responder side, and iii) plants kept untreated on the inducer side and inoculated with J2 on the responder side (control). Bacteria were applied by drenching 20 ml of a bacterial suspension (OD_560_ = 2) into holes made at the inducer side. Three days later, 1,000 J2 in 2 ml water were inoculated into holes made at the respective pot side. Each treatment was replicated ten times and arranged in a randomized block design in the greenhouse. A duplicated setup of the experiment was sacrificed after seven days to evaluate J2 penetration into roots as described above. After 50 days the numbers of galls, egg masses, and eggs per plant were determined.

### Statistical analysis

Analysis of variance was done using the procedure GENMOD of the statistical software SAS 9.3 (SAS Institute Inc., Cary, NC, USA) to fit generalized linear models. For count data (numbers of galls, egg masses, eggs, J2 in roots) the procedure was used to perform a Poisson regression analysis with a log link function and specification of a scale parameter (Pearson) to fit overdispersed distributions. Class variables were treatment (strain or uninoculated control) and block (accounting for the randomized block design of experiments). For multiple comparisons of strain effects the *p*-value was adjusted by the method of Tukey. Repellence (experiment 3) was statistically tested using the procedure GENMOD as explained to compare the numbers of J2 in roots at the uninoculated side of the linked twin-pot system between treatments. The effect of the different strains on growth of plants infected by *M. incognita* was tested by MANOVA using the SAS procedure GLM, with the dependent variables root weight, root length, shoot weight, shoot length, and number of leaves. For multiple comparisons of the effect of antagonistic strains to the *E. coli* control the *p*-value was adjusted by the method of Dunnett.

## Results

### Potential of seed-inoculated strains to control *M. incognita*

In total nine bacteria were tested for their antagonistic potential towards *M. incognita* by seed inoculation (experiment 1). The number of galls and egg masses developed by *M. incognita* was highest in the non-inoculated control and the treatment with the non-antagonistic strain *E. coli* JM109 (Table 2). Significantly less galls and egg masses than in these controls were found in the treatments with the biocontrol strains, except for Sb3-24 and 3Rc2-7. The highest control potential was achieved by strain Sb4-23, which did not significantly differ from the well studied positive control G12. It caused 86% reduction in the number of galls and 96% reduction in number of egg masses compared with the untreated control. Good biocontrol was also achieved by the two other *Bacillus subtilis* isolates Mc2-Re2 and Mc5-Re2 with over 60% reduction in number of galls and over 70% reduction in number of egg masses. Based on these results, the isolates Sb4-23, Mc2-Re2, and Mc5-Re2 were selected for studying their mode of action in nematode suppression.

**Table 2:** Effect of bacterial seed treatment on number of galls and egg masses of *M. incognita* after propagation on tomato plants.

Bacterial inoculant	Galls per plant (± stdev)	Treatment effect on no. of galls a			Egg masses per plant (± stdev)	Treatment effect on no. of egg masses a	
Culture medium	331±35		A		269±38		A
*E. coli* JM109	316±39	B	A		193±48		B
Sb3-24	267±87	B	A	C	164±64		B
3Rc2-7	240±58	B	D	C	135±37	C	B
C48	195±48		D	C	104±31	C	D
Ru47	185±62	E	D		76±32		D
Mc2-Re2	122±73	E	F		70±47	E	D
Mc5-Re2	80±27	G	F		35±17	E	F
G12 (+ control)	48±25	G			12±10	G	F
Sb4-23	45±24	G			11±14	G	

a Tukey-Kramer grouping for least squares means (α = 0.05): Means followed by the same letter are not significantly different (n = 12).

### Effect of bacterial culture supernatants on *M. incognita*

The isolates Sb4-23, Mc5-Re2, and Mc2-Re2 selected from experiment 1 were tested for negative effects of their metabolites on *M. incognita* (experiment 2). Application of cell-free culture supernatants of all three tested strains and the positive control G12 significantly reduced the number of galls, egg masses, and eggs on tomato roots compared to the treatments with *E. coli* culture supernatant or sterile culture medium (Table 3). The lowest average number of galls was observed in the Sb4-23 treatment, which did not significantly differ from Mc2-Re2 and the positive control G12 but from Mc5-Re2. Among the bacterial antagonists, no differences were observed in numbers of egg masses and eggs per root. The number of eggs per egg mass was significantly lower for the treatments with Sb4-23 and G12 metabolites than for the negative controls.

**Table 3:** Effect of bacterial culture supernatants on reproduction of *M. incognita* on tomato plants.

Applied culture	Average no. per plant ± stdev. a							
supernatant	Galls		Egg masses		Eggs (x 1,000)		Eggs/egg mass	
Culture medium	172±14	A	129±16	A	41±6	A	322±67	A
*E. coli* JM109	136±16	B	98±15	A	32±7	A	330±67	A
Mc5-Re2	98±20	C	67±22	B	19±6	B	282±31	AB
G12	83±20	CD	60±18	B	14±7	B	224±41	C
Mc2-Re2	80±13	CD	49±11	B	13±3	B	275±49	ABC
Sb4-23	75±17	D	54±21	B	13±5	B	253±23	C

a Tukey-Kramer grouping for least squares means: Means followed by the same letter within each column are not significantly different (α = 0.05, n = 10).

The culture supernatants of the strains significantly differed in their effect on plant growth during nematode exposure, as revealed by MANOVA of the length and weight of root and shoot, and the number of leaves 50 days after nematode inoculation (P = 0.005, Table 4). Among the three strains tested, only metabolites of Mc2-Re2 significantly enhanced plant growth compared to the *E. coli* control, as evidenced by increased root length (P = 0.006, Dunnett test) and number of leaves (P = 0.03). G12 had a positive effect on root length.

**Table 4:** Effect of bacterial culture supernatants on plant growth of tomato infected with *M. incognita.*

Applied culture supernatant	Root		Shoot		No. of leaves
	Length (cm)	Weight (g)	Length (cm)	Weight (g)	
Culture medium	12.1±1.7	2.9±0.5	33.8±3.8	11.4±1.5	8.5±1.3
*E. coli* JM109	12.9±1.3	3.2±0.7	35.5±1.8	11.9±0.8	8.8±0.8
Mc5-Re2	14.1±1.9	3.2±0.5	39.1±3.8	12.6±0.8	9.4±0.7
Sb4-23	14.1±1.4	3.3±0.4	37.2±4.4	11.6±0.9	9.6±0.8
G12	14.8±1.4 *	3.5±0.6	34.0±2.0	12.2±0.8	9.5±0.7
Mc2-Re2	15.0±1.1 *	3.1±0.6	38.0±3.6	12.0±1.1	9.7±0.7 *

^*^ Significantly different (*P*≤0.05, Dunnett adjustment, n = 10) to both control treatments (JM109 culture supernatant and sterile culture medium).

### Effect of antagonistic strains on repellence of J2

A linked twin-pot set-up was used to evaluate the effect of bacterial antagonists on attraction of *M. incognita* J2 to tomato roots (experiment 3, Fig. 1A). One week after inoculating the nematodes at the centre of a tube connecting two pots planted with tomato, the numbers of J2 that moved to one or the other side and penetrated the roots were counted (Fig. 1B). As a trend, slightly more J2 were found in the roots at the uninoculated side of linked twin-pot systems that were treated with biocontrol strains compared to the treatment with *E. coli* or the control. However, the difference was not statistically significant (P = 0.10). None of the treatments with biocontrol strains significantly differed from that with *E. coli*. Pots which were inoculated with biocontrol strains showed a trend for less penetrated J2 in the roots compared to the linked uninoculated pots.

### Induced systemic resistance towards *M. incognita*

To test the potential of bacterial antagonists to induce systemic resistance, bacteria and *M. incognita* were applied spatially separated on tomato roots within a split-root system (experiment 4, Fig. 2A). The treatment on the inducer side had a significant effect on the number of galls and egg masses on the responder side (P < 0.0001). In split-root systems with the three tested biocontrol strains or G12 significantly less galls and egg masses were detected 50 days after inoculation of the nematodes compared to the untreated control or plants treated with *E. coli* (Fig. 2B). The number of galls or egg masses was 40% to 51% lower in treatments with the biocontrol strains. The highest reduction on average was obtained by the strains Mc2-Re2 and Mc5-Re2, but differences between the four antagonistic bacteria were not significant. The negative control *E. coli* was not different from the uninoculated control, thus induction of resistance was not detectable for this non-antagonistic bacterium.

### Plant-mediated rather than direct effect of biocontrol strains on *M. incognita*

**Figure 3:**
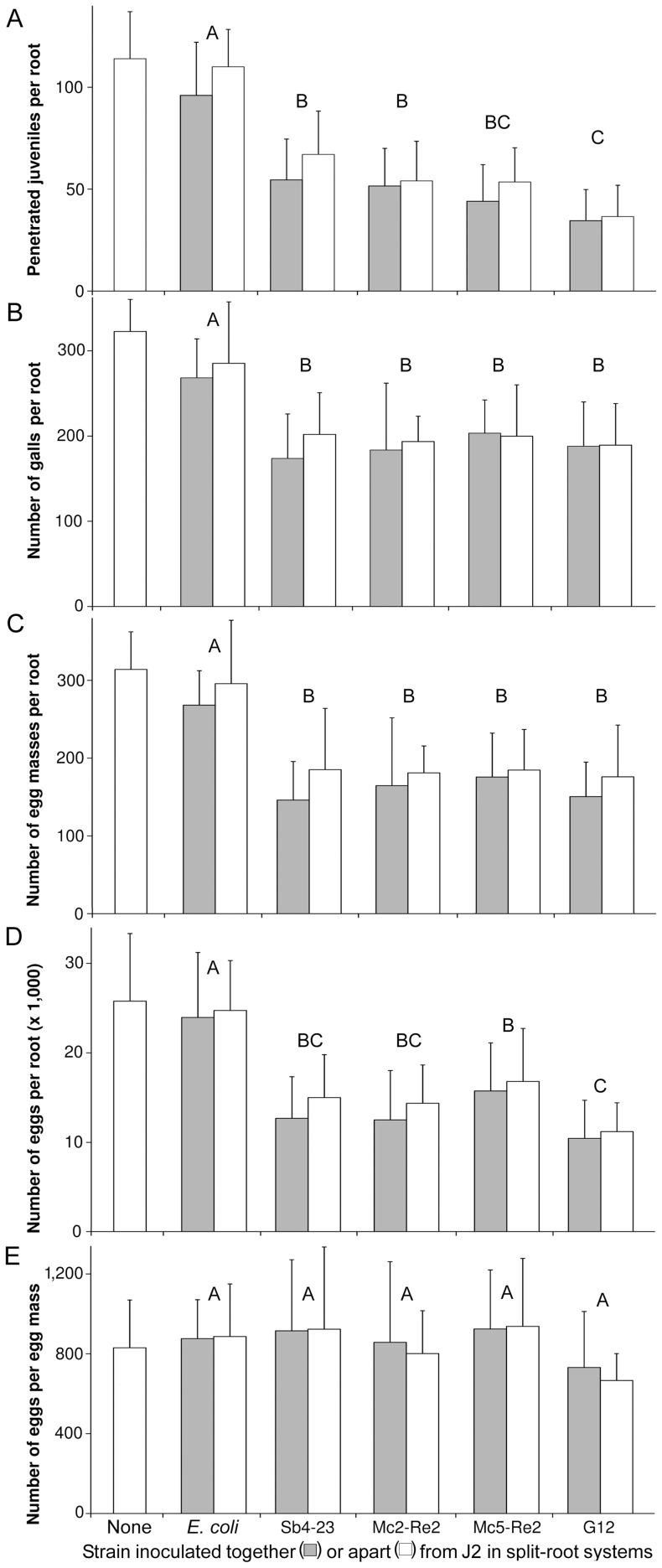
Comparison of the effects by direct and by plant-mediated antagonism on root penetration by juveniles. *M. incognita* juveniles (J2) and bacterial strains were inoculated spatially separated in opposite pots of one split-root system (white bars), or co-inoculated with J2 in one pot of another split root system (gray bars). Controls were inoculated with J2 and the not antagonistic strain *E. coli* JM109, or only with nematodes. J2 penetrated into tomato roots were counted 10 days after inoculation (A). Numbers of galls (B), egg masses (C), eggs per root (D), and eggs per egg mass (E) were determined 50 days after J2 inoculation. Error bars represent standard deviations. Different letters indicate significant differences at *P≤0.05* according to Tukey's test (n = 10).

In split-root systems the plant-mediated effect of the bacteria on *M. incognita* was compared to the combined plant-mediated and direct effect when bacteria and J2 are co-inoculated in the same pot (experiment 5). One week after nematode inoculation, all antagonistic bacteria significantly reduced J2 penetration compared to the negative control *E. coli*, which did not differ from the control without inoculated bacteria (Fig. 3A). The lowest numbers of penetrated juveniles were observed for G12 and Mc2-Re2, corresponding to of 67% and 52% reduction compared to the control, respectively. Three-factorial analysis of variance revealed a significant difference between stains in their effect on root penetration of J2 (P < 0.0001), and a significant decrease of J2 by co-inoculation with bacteria (P = 0.01). However, J2 in roots were only slightly decreased by co-inoculation of J2 and biocontrol strains, so that most of the biocontrol effect on J2 can be explained by induced systemic resistance alone.

Fifty days after nematode inoculation, in all treatments with bacterial antagonists significantly less galls, egg masses, and eggs were found compared to the treatment with *E. coli*, or the untreated control (Fig. 3B-D). Three-factorial analysis of variance revealed a significant difference between stains in their effect on nematode reproduction (P < 0.0001). Co-inoculation did not have a detectable effect on numbers of galls or eggs (P = 0.3 or 0.2, respectively), and only a slight effect on egg masses (P = 0.049). Thus, most of the biocontrol effect on reproduction can be explained merely by induced systemic resistance. The three tested biocontrol strains did not significantly differ in their potential to suppress *M. incognita*. The positive control strain G12 could slightly better reduce the number of eggs compared to strain Mc5-Re2 in this experiment. No significant effect of the bacteria on the number of eggs per egg mass was detected (Fig. 3E).

## Discussion

Within this study seven antagonistic bacteria with known antagonism towards fungal pathogens were selected and tested for their potential to control *M. incognita* on tomato. Five of the bacterial antagonists significantly reduced *M. incognita* infestation on tomato after seed treatment. It was shown that individual bacterial antagonists have a much broader control spectrum than originally thought by concomitantly controlling fungal pathogens and plant-parasitic nematodes. The results are in accordance with previous work where potato-associated strains of *Pseudomonas* and *Streptomyces* inhibited both the soil-borne fungal wilt pathogen *V. dahliae* and the root-knot nematode *M. incognita* [8]. Similarly, Tariq et al. [9] were able to show that a strain of *P. aeruginosa* inhibited both the root-rotting fungi *M. phaseolina*, *R. solani*, *F. solani*, and *F*. *oxysporum* as well as the root-knot nematode *M. javanica* infecting chili roots.

In the present study nematode antagonism was shown for strains belonging to the species *B. subtilis*, *P. jessenii*, and *S. plymuthica*. All antagonistic bacteria were able to significantly reduce galls and egg masses on tomato compared with the untreated control. While other strains of *B. subtilis* and *S. plymuthica* have been reported as nematode antagonists before [22]–[25], strains with biocontrol potential belonging to the species *P. jessenii* were first reported in this study. The positive control *R. etli* G12 confirmed its good biocontrol potential [26]. Within experiment 1, bacterial isolates were applied as a seed treatment. The good results achieved by this method raises optimism that seed treatment could be an efficient and economical way for bacterial delivery in practise as already reported for other bacterial antagonists [27], [28].

Besides seed treatment also a soil drench with culture supernatants of the antagonistic bacteria resulted in a significant reduction in galls, egg masses, and eggs produced by *M. incognita*. Nematode suppression by bacterial culture supernatants has previously been reported when testing for antibiosis under *in vitro* conditions [29], [30]. Unfortunately, still very little is known about the active compounds of culture supernatants causing nematode antagonism. Siddiqui et al. [31] found that for *P. aeruginosa* the ethyl acetate extract caused 64% inactivity of *M. javanica* juveniles within 24 h and assumed that the active compound was of proteinaceous or glycoproteinaceous nature. The active compound was described as heat sensitive, sensitive to extreme pH values, polar in nature and with a molecular weight smaller than 8,000 Da [32].

Padgham and Sikora reported that *Bacillus megaterium* caused repellence of *Meloidogyne graminicola* from rice roots [12]. Production of repellent substances or modification of the plant’s exudates by the antagonistic bacteria were suggested as mechanisms for this effect [10]. In our study, a trend for repellence of *M. incognita* by the tested biocontrol strains was observed, although it was not statistically significant due to high variation between replicates. A complete different mechanism involved in bacteria-mediated nematode control is induced systemic resistance of the plant. In relation to nematode control, induced systemic resistance was first reported by Hasky-Günther and Sikora [33]. In our study using a split-root system, all four antagonistic bacteria tested induced systemic resistance towards *M. incognita* in tomato. Galls and egg masses were reduced between 40% and 51%, respectively, which was in the range of control rates reported for similar studies [34]–[36]. For the positive control strain *R. etli* G12 used in the present study it was shown that viable as well as dead bacterial cells were able to trigger the systemic resistance response in potato against the potato cyst nematode *Globodera pallida*. Furthermore, it turned out to be the oligosaccharides of the core-region of the bacterial lipopolysaccharides to be the main trigger of the resistance response [36].

Our experimental setup allowed for the first time to compare between the plant-mediated antagonistic effect of the strains and direct effects of the bacteria on the nematode caused by nematicidal, nematostatic or repellant bacterial compounds or parasitism on juveniles or eggs. In comparison with induced systemic resistance the application of the antagonistic bacteria together with the nematodes on the responder side of the split-root system only slightly enhanced the biocontrol effect. Thus induced systemic resistance was identified as the the major control mechanism of the antagonists in this study (experiment 5). For all tested strains bacterial cells and cell-free culture supernatants caused similar reductions in galls, egg masses, and eggs. Together with the just mentioned result of experiment 5, this suggested that systemic resistance in tomato was induced by compounds from the bacteria that can also be found in the culture supernatants.

In conclusion, all bacterial antagonists with known antifungal capacity tested in this study also showed antagonistic activity against the root-knot nematode *M. incognita*. The control potential of the three *B. subtilis* strains Sb4-23, Mc2-Re2, and Mc5-Re2 was within the range of the positive control *R. etli* G12. For all tested strains seed treatment with bacterial cells as well as bacterial culture supernatants caused similar reductions in number of galls, number of egg masses and total number of eggs per plant. The results achieved with *B. subtilis* were especially stimulating since it produces spores that are a lot easier to formulate and store than Gram-negative bacteria such as *R. etli* G12 or the tested *Pseudomonas* strains. Overall best nematode control in this study was achieved by *B. subtilis* Sb4-23 making this isolate a promising candidate for dual biocontrol of *M. incognita* and seed-borne fungal pathogens under field conditions.
